# Hyperprolactinémie: coexistence rare d'une hypothyroïdie périphérique et d'un microprolactinome

**DOI:** 10.11604/pamj.2016.24.41.8537

**Published:** 2016-05-10

**Authors:** Asmaa Chafik, Ghizlane El Mghari, Nawal El Ansari

**Affiliations:** 1 Service d'Endocrinologie Diabétologie et Maladies Métaboliques, Hopital Arrazi, CHU Mohamed VI, Faculté de Médecine et de Pharmacie, Université Cadi Ayad, Marrakech, Maroc

**Keywords:** Hyperprolactinémie, prolactinome, hypothyroïdie, Hyperprolactinemia, prolactinoma, hypothyroidism

## Abstract

Nous rapportons un cas rare d'hyperprolactinémie révélant l'association d'une hypothyroïdie périphérique à un micro adénome hypophysaire à prolactine. Il s'agit d'une patiente âgée de 43 ans, consultant pour galactorrhée bilatérale spontanée depuis 1an. L'hyperprolactinémie a été confirmée, avec au bilan étiologique une hypothyroïdie périphérique secondaire à une thyroïdite auto-immune. La prise en charge a consisté la mise sous hormone thyroïdienne, avec une stabilisation clinique et normalisation hormonale 3 mois plus tard. L’évolution a été marquée par la persistance de la galactorrhée avec hyperprolactinémie. Le diagnostic de microprolactinome a été retenu devant l'image de microadénome à l'IRM hypophysaire, justifiant la mise sous traitement anti dopaminergique. Six mois plus tard, l’évolution a été marquée par la normalisation du taux de prolactine et disparition de l'image de microadénome hypophysaire.

## Abstract (en)

We report a rare case of hyperprolactinemia revealing the association between peripheral hypothyroidism and prolactin pituitary macroadenomas. The patient was a 43-year old woman, presenting with spontaneous bilateral galactorrhea over a period of 1 year. Hyperprolactinemia was confirmed and etiologic investigation revealed peripheral hypothyroidism secondary to autoimmune thyroiditis. Therapy consisted of administration of thyroid hormone, with clinical stabilization and hormonal normalization three months later. The evolution was marked by the persistence of hyperprolactinemia and galactorrhea. The diagnosis of microprolactinoma was objectified by pituitary MRI which showed microadenoma, justifying the administration of antidopaminergic therapy Six months later, the evolution was marked by normalization of prolactin levels and disappearance of pituitary microadenoma image.

## Introduction

L’ hyperprolactinémie représente une situation clinique fréquente, et cause de 20 à 25% des aménorrhées secondaires [1]. Elle se définit par une élévation de la concentration plasmatique de prolactine au-delà de la limite supérieure du dosage. Ainsi une démarche diagnostique orientée par la sémiologie clinique, permet un diagnostic étiologique bien précis. Les adénomes à prolactine constituent la cause la plus fréquente d'hyperprolactinémie non médicamenteuse, avec une sécrétion tumorale de prolactine. Mais certaines pathologies comme l'hypothyroïdie périphérique, l'insuffisance rénale ou l'insuffisance hépatique sévère doivent être considérées et éliminées au préalable. En cas d'hypothyroïdie périphérique, des cas sont rapportés décrivant une hyperplasie hypophysaire homogène [2, 3]. Nous rapportons à travers cette observation une coexistence rare d'hypothyroïdie périphérique et micro adénome hypophysaire, et discutons le lien de causalité à l'hyperprolactinémie entre ces deux atteintes.

## Patient et observation

**Figure 1:**
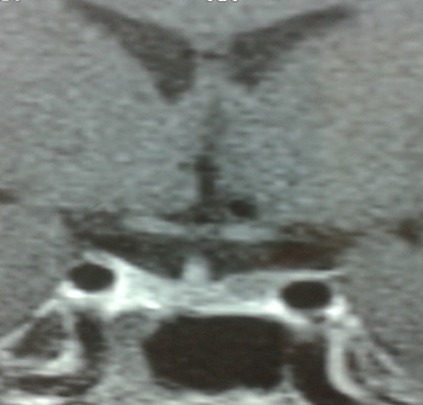
Coupe sagittale d'IRM hypophysaire avec image de micro adénome hypophysaire gauche

**Figure 2:**
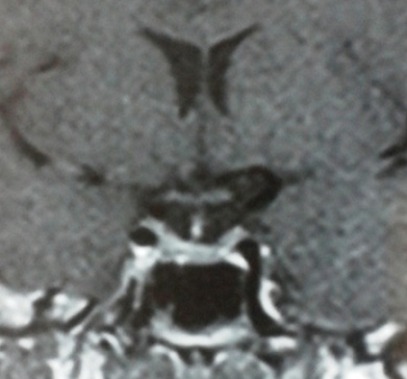
Disparition d'image d'adénome hypophysaire après traitement

Nous rapportons l'observation d'une patiente âgée de 43ans, consultant pour galactorrhée bilatérale spontanée depuis 1 an. L'interrogatoire retrouve la notion de céphalées rétro orbitaires intermittentes d'intensité modérée évoluant depuis 3 ans, sans baisse de l'acuité visuelle, avec une spanioménorrhée évoluant depuis 2ans, le tout associé à une dépilation et constipation évoluant depuis 2ans, sans notion de prise médicamenteuse. L'hyperprolactinémie a été confirmé par deux dosages à 86ng/ml et 82ng/ml. Le diagnostic d'hypothyroïdie périphérique a été posé devant une TSH élevée à 45mUI/ml avec une T4 libre basse à 10,77 pmol/l, avec au bilan étiologique échographique une thyroïde hétérogène siège de multiple plages hypoéchogènes avec hyper vascularisation au doppler compatible avec un aspect de thyroïdite et un titre élevé d'anticorps antithyropéroxydase à 132mUI/l. Le bilan hépatique et rénal était normal. L'IRM hypothalamo-hypophysaire a objectivé un micro adénome hypophysaire de 3.5mm à gauche avec un léger bombement du diaphragme sellaire (Figure 1) sans retentissement visuel. La prise en charge a consisté en la mise sous hormones thyroïdiennes, avec une évaluation clinique et hormonale 3 mois après. L’évolution a été marquée par la persistance de la galactorrhée bilatérale avec spanioménorrhée, malgré la normalisation du bilan thyroïdien avec une TSH à 2.03 mUI/l et une T4 libre à 14pmol/l et une prolactinémie toujours élevée. Par conséquent, le microadénome hypophysaire a été considéré comme cause d'hyperprolactinémie, justifiant la mise sous traitement anti dopaminergique cabergoline à la dose de 0,5mg par semaine, que la patiente a observé pendant 6mois. L’évolution a été marquée par la normalisation du taux de prolactine et disparition de l'image de microadénome hypophysaire (Figure 2) après 6mois de traitement antidopaminergique.

## Discussion

L'hyperprolactinémie est l'anomalie hormonale endocrinienne la plus fréquent dans l'atteinte de l′axe hypothalamo-hypophysaire, ses causes étant très nombreuses: médicamenteuses, atteintes hypophysaires, maladies générales, hypothyroïdie. Les adénomes à prolactine, tumeurs bénignes dues à la prolifération de cellules lactotropes de l'hypophyse représentent la cause la plus fréquente des adénomes hypophysaires [4]. Leur pathogénie est incomplètement connue; certaines anomalies moléculaires, dont quelques protooncogènes (ras, PTTG) ou des facteurs de croissance et leurs récepteurs (NGF, FGFR) ont été mise en évidence dans quelque cas, sans que pour l'instant un modèle ne puisse être proposé pour le développement de ces tumeurs [5]. Les études familiales ont permis d'impliquer le gène des néoplasies endocriniennes multiples (NEM) de type 1 et le gène récemment décrit AIP [6]. Dans l'hypothyroïdie périphérique, l'hyperprolactinémie peut être expliquée par plusieurs mécanismes. Tout d′abord, les niveaux de prolactine peuvent être attribués à l’élévation de la TRH (thyrotropin releasing hormone) qui est un stimulateur hypothalamique aussi bien des cellules lactotropes que thyréotropes; la clairance de la prolactine peut aussi être diminuée chez les hypothyroïdiens [7]. La réduction de la sensibilité de prolactine à l′action inhibitrice de la dopamine et des agonistes dopaminergiques en présence d'une hypothyroïdie est suggérée par Foord et al. [8], plusieures séries de cas rapportent la normalisation des taux sériques de prolactine après le traitement par L-thyroxine chez les patients atteints d′hypothyroïdie [9]. Néanmoins, chez notre patiente, l’évolution sous traitement substitutif thyroïdien a permis de normaliser le bilan thyroïdien, mais avec persistance de galactorrhée et de l'hyperprolactinémie. Ainsi, l'hypothèse de la coexistence d'un microprolactinome est fort probable, d'autant plus que la prolactinémie s'est normalisé après traitement antidopaminergique avec régression totale de l'image de microadénome. Cependant, l'hyperprolactinémie peut être la résultante de deux étiologies de physiopathologie différente; des études récentes ont dans ce sens démontré son fort effet stimulant immunitaire, avec des effets modulateurs immunitaires interférant avec la tolérance des lymphocytes B, augmentant ainsi la production des cytokines. L'hyperprolactinémie est par ailleurs observée dans diverses maladies auto-immunes notamment dans le lupus, la sclérose en plaques et le syndrome de Sjogren [10]. Par conséquent, nous pouvons éventuellement supposer que l'hyperprolactinémie induite par le prolactinome à favorisé l'auto-immunité thyroïdienne, qui a entretenu en elle-même l'hyperprolactinémie par un mécanisme physiopathologique différent.

## Conclusion

Cette observation illustre le défi étiologique devant l'association aléatoire de deux atteintes, devant lesquelles il faut argumenter la prise en charge adéquate qui cible l’étiologie à l'origine de la symptomatologie. Bien que l'hyperprolactinémie puisse être la résultante de deux étiologies différentes, un lien physiopathologique entre ces deux atteintes ne peut être exclu, que ça soit par le rôle immuno-stimulateur de la prolactine dans le déclenchement de l'atteinte auto-immune thyroidienne, ou par la possibilité d'une entité de prédisposition génétique à ces deux atteintes dans une nouvelle entité syndromique.
